# Clinical Scores of Peripartum Patients Admitted to Maternity Wards Compared to the ICU: A Systematic Review and Meta-Analysis

**DOI:** 10.3390/jcm14145113

**Published:** 2025-07-18

**Authors:** Jennifer A. Walker, Natalie Jackson, Sudha Ramakrishnan, Claire Perry, Anandita Gaur, Anna Shaw, Saad Pirzada, Quincy K. Tran

**Affiliations:** 1 Department of Emergency Medicine, Baylor Scott & White All Saints Medical Center, Fort Worth, TX 76104, USA; 2 Burnett School of Medicine, Texas Christian University, Fort Worth, TX 76104, USA; 3 Department of Obstetrics and Gynecology, Baylor Scott & White All Saints Medical Center, Fort Worth, TX 76104, USA; natalie.jackson@bswhealth.org; 4 Baylor Health Science Library, Dallas, TX 75246, USA; sudha.ramakrishnan@bswhealth.org; 5 Research Associate Program in Emergency Medicine and Critical Care, Department of Emergency Medicine, University of Maryland School of Medicine, Baltimore, MD 21201, USA; cperry32@jhu.edu (C.P.); anangaur@terpmail.umd.edu (A.G.); annaes7302@gmail.com (A.S.); saadpirzada147@gmail.com (S.P.); qtran@som.umaryland.edu (Q.K.T.); 6 Department of Emergency Medicine, University of Maryland School of Medicine, Baltimore, MD 21201, USA; 7 Program in Trauma, The R Adam Cowley Shock Trauma Center, University of Maryland School of Medicine, Baltimore, MD 21201, USA

**Keywords:** pregnancy, high risk, critical care medicine, triage, outcomes

## Abstract

**Background/Objectives**: Hospitalized peripartum patients who later decompensate and require an upgrade to the intensive care unit (ICU) may have an increased risk for poor outcomes. Most of the literature regarding the need for ICU involves Modified Early Warning Scores in already hospitalized patients or the evaluation of specific comorbid conditions or diagnoses. This systematic review and meta-analysis aimed to assess the differences in clinical scores at admission among adult peripartum patients to identify the later need for ICU. **Methods**: We systematically searched Ovid-Medline, PubMed, EMBASE, Web of Science and Google Scholar for randomized and observational studies of adult patients ≥18 years of age who were ≥20 weeks pregnant or up to 40 days post-partum, were admitted to the wards from the emergency department and later required critical care services. The primary outcome was the Sequential Organ Failure Assessment (SOFA) score. Secondary outcomes included other clinical scores, the hospital length of stay (HLOS) and mortality. The Newcastle–Ottawa Scale was utilized to grade quality. Descriptive analyses were performed to report demographic data, with means (±standard deviation [SD]) for continuous data and percentages for categorical data. Random-effects meta-analyses were performed for all outcomes when at least two studies reported a common outcome. **Results**: Seven studies met the criteria, with a total of 1813 peripartum patients. The mean age was 27.2 (±2.36). Patients with ICU upgrades were associated with larger differences in mean SOFA scores. The pooled difference in means was 2.76 (95% CI 1.07–4.46, *p* < 0.001). There were statistically significant increases in Sepsis in Obstetrics Scores, APACHE II scores, and HLOS in ICU upgrade patients. There was a non-significantly increased risk of mortality in ICU upgrade patients. There was high overall heterogeneity between patient characteristics and management in our included studies. **Conclusions**: This systematic review and meta-analysis demonstrated higher SOFA or other physiologic scores in ICU upgrade patients compared to those who remained on the wards. ICU upgrade patients were also associated with a longer HLOS and higher mortality compared with control patients.

## 1. Introduction

Early Warning Scores (EWS) are used in hospitalized patients to determine if they are at risk for clinical decline and require an upgrade to higher-level care [1,2,3,4,5,6]. EWS are typically not generalizable to the obstetric population due to physiologic changes in pregnancy; therefore, work has been conducted to discover and validate various Modified EWS for obstetric and peripartum patients [7,8,9,10,11,12].

Patients who are initially admitted to the ward and then require transfer to the ICU have worse outcomes [13,14,15] than those who do not require ICU upgrades. Wardi et al., for example, describe that, even with less severe indices of illness at presentation, the mortality of patients who upgraded to the ICU was much higher than in those who remained on the general wards (25% versus 8%) [14]. Besides an increased mortality risk for patients requiring unexpected ICU upgrades, these unexpected ICU upgrades also use scarce critical care resources. It is important to utilize scarce and costly critical care resources diligently, and this may be achieved by accurately admitting patients with a risk of ICU upgrade to the ICU initially. Inappropriate use of critical care resources when patients are “too well” is also a concern from a financial as well as staff morale standpoint [16,17]. It is imperative, therefore, to recognize patients at admission and appropriately triage them to reduce morbidity and mortality and improve financial metrics and morale.

There is an abundance of literature looking at higher-risk peripartum patients whose demographic information and comorbidities indicate risks for ICU admission. Many of these studies examine which obstetrical diagnoses are most commonly admitted to the ICU, as well as comorbidities such as tobacco use, hypertension or age [18,19,20,21,22,23]. Most of these studies do not have a control group of pregnant patients who did not require ICU upgrades, and these studies focus on diagnoses and comorbidities rather than objective clinical scores at admission.

For non-pregnant patients, there are a few scoring systems used in the emergency department setting to help determine appropriate disposition and admission locations for patients, such as the CURB-65 for pneumonia or SOFA for sepsis [24,25]. Again, many of these scoring systems have not been well studied in the obstetric population and do not account for the physiologic changes in pregnancy. This can make it challenging for emergency providers to determine appropriate admission levels for these patients. Early identification and treatment are key components in preventing maternal mortality.

Since a limited number of studies have looked at specific clinical scores in pregnancy to help determine the risk for decompensation at admission and the need for critical care services, this review aims to identify clinical scores to better triage obstetric patients to appropriate clinical settings.

## 2. Materials and Methods

### 2.1. Eligibility Criteria, Information Sources, Search Strategy and Selection Process

This systematic review and meta-analysis was conducted by following a pre-specified protocol that was based on the guidelines outlined in the Preferred Reporting Items for Systematic Reviews and Meta-Analyses (PRISMA) statement [26].

We systematically searched Ovid-Medline, PubMed, EMBASE, Web of Science and Google Scholar. A copy of our search strategy is provided in Appendix A.

This protocol included randomized trials and observational (prospective or retrospective) studies of adult patients ≥18 years of age who were ≥20 weeks pregnant or up to 40 days post-partum, who were admitted to the wards from the emergency department. The protocol excluded pediatric patients and patients admitted to the hospital from sources other than the emergency department (i.e., interhospital direct transfers). Also excluded were patients less than 20 weeks pregnant or greater than 40 days post-partum, those with previously known abnormal pregnancies or direct admissions to the ICU. Literature reviews, systematic reviews, meta-analyses, letters to the editor, abstracts, other “grey literature” and animal studies were excluded.

This protocol was registered with PROSPERO (CRD42023488092). Our database searches were performed in November 2023, with citation searching in June 2024. Prior to the completion of the final data analysis, a final database search through January 2025 was performed. No date or language limits were placed on these searches, and forward and backward citations were screened for included studies.

There were deviations from the pre-specified protocol due to issues with heterogeneity in the included populations, exclusion criteria and outcome reporting. Many studies did not consistently list the patient age, gestational age or whether there were pre-existing pregnancy complications as an exclusion criterion. We included these studies if there were no explicit exclusion criteria noted. Moreover, because most studies did not look at clinical scores specifically at admission, we used a cutoff of 48 h for outcome measurements of clinical scores. Lastly, while we hoped to look at studies of patients admitted to the ward who later required an upgrade to the ICU, some studies did compare the ED scores of patients who required the ICU at admission. Since our group determined that these studies would reflect our specific clinical question as to whether there were clinical scores that would reflect patients needing critical care, we decided to include these studies. Specific protocol deviations in each study, if indicated, are included in Appendix B.

### 2.2. Outcome Measures

Our primary outcome was the Sequential Organ Failure Assessment (SOFA) score. Secondary outcomes included Acute Physiology and Chronic Health Evaluation (APACHE) scores and other clinical scoring systems. Hospital stays (HLOS) and mortality were also recorded if available. These outcomes were compared to whether the patient required ICU-level care. We reported pooled outcomes if at least two studies reported on them.

### 2.3. Study Selection and Data Extraction

We assigned a total of 7 reviewers for the initial screening process (JAW, NJ, QKT, CP, AG, SP, AS), and three of these were senior investigators (JAW, NJ, QKT). Two investigators independently reviewed each title and abstract, but at least one senior investigator was required to screen each title and abstract to ensure consistency. If there was discordance, a second senior investigator was the tiebreaker. Each abstract required at least 2 agreements to move forward to the full-text screening stage. This same process was applied to the full-text screening process.

All abstracts and titles that met the initial criteria were further reviewed as full-text articles, with a similar review process as noted above. Studies that met the eligibility criteria were included in the final analysis. Covidence was utilized as a screening management system (Covidence systematic review software, Veritas Health Innovation, Melbourne, Australia).

Data from the included studies were extracted, including the study name, study author and year, study design, patient sample size, study setting, inclusion and exclusion criteria, primary outcome, secondary outcomes, patient demographics and other clinical parameters, such as vital signs and laboratory markers.

If total population means were not reported, they were calculated based on means for the ICU and non-ICU samples. The shock index was also calculated for some studies based on the provided clinical data. The data were extracted into a standardized Excel spreadsheet (Microsoft Corp., Redmond, WA, USA), with all three senior investigators evaluating the extracted data to confirm their accuracy.

### 2.4. Quality Assessment and Heterogeneity

The Newcastle–Ottawa Scale (NOS) was utilized to assess the quality of observational studies [27]. The NOS assesses 3 domains (sample selection, group comparability and quality of outcomes). Studies are awarded a maximum of 9 points. High-quality studies score ≥ 7, whereas moderate-quality studies have scores of 4–6 and low-quality studies have scores of ≤3. Two investigators independently applied the NOS. Disagreements were resolved by discussion between these two investigators (AG and AS) and the lead investigator (JW). The final results were reported as the group consensus among those who were involved. Heterogeneity was assessed with I^2^ values.

### 2.5. Statistical Analysis

This study used descriptive analyses to report demographic data, with means (±standard deviation [SD]) for continuous data and percentages for categorical data. For studies that reported the median and interquartile range [IQR], we converted the median to the mean and SD by using an online calculator [vassarstats.net]. Random-effects meta-analyses were performed for all outcomes when at least 2 studies reported a common outcome. We reported the results from meta-analyses with continuous outcomes as mean differences and their associated 95% confidence intervals (95% CI) and *p*-values. For the categorical outcome of mortality, the results were expressed as the log odds ratio and 95% CI. We chose the log odds ratio because ICU-upgraded patients were associated with overwhelming odds of morality, resulting in a very high odds ratio. Furthermore, due to the small number of included studies, we did not perform moderator analyses to assess sources of heterogeneity or differences between subgroups of patients. Similarly, we did not create funnel plots for publication bias, although Egger’s and Begg’s tests were performed to identify publication bias for any outcome analyses containing at least three studies, as these tests are unreliable if only two studies are included. We did not perform a sensitivity analysis. Meta-analyses and publication bias analyses, when appropriate, were performed with the software Comprehensive Meta-Analyses (CMA version 4.0, www.meta-analysis.com, Englewood, NJ, USA). All statistical comparisons with *p*-values < 0.05 were considered statistically significant

## 3. Results

### 3.1. Study Selection

**Figure 1:**
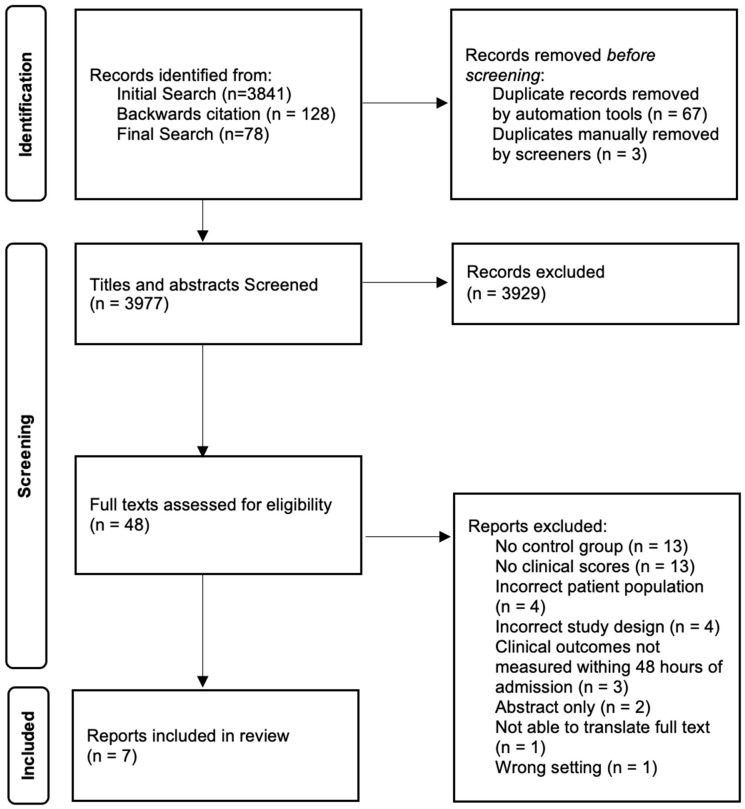
PRISMA 2020 diagram for study identification and selection.

Our initial search on 9 November 2023 identified 3841 studies. An additional 128 studies were included through backwards citation after the included citations were screened for any additional relevant references. Prior to manuscript drafting, a final search was included in Covidence for screening, with 78 references added on 24 January 2025. After the removal of duplicates, a total of 3977 titles and abstracts were screened for inclusion. After the completion of the full-text evaluation with protocol deviations, a total of seven studies were included (Figure 1).

### 3.2. Study Characteristics

All seven studies were observational and were published between 2014 and 2025. Table 1 describes the characteristics of the included studies. Three studies (43%) were retrospective [28,29,30], and there were four (57%) prospective cohort studies [31,32,33,34]. Four studies compared Sequential Organ Failure Assessment (SOFA) scores [29,30,32,33], which was our primary outcome. The majority of studies examined sepsis in peripartum patients (71%).

Concerning the scores analyzed, the Sepsis in Obstetrics Score (SOS) was evaluated most frequently [28,31,32,33,34]. These studies were included as they did meet the criteria for the evaluation of clinical scores of patients at admission. Lastly, Minville et al. studied several clinical scores, including the SAPS II, SOFA and APACHE II; however, this study used a marker of the nursing workload—the Therapeutic Intervention Scoring System (TISS 28)—rather than upgrade to critical care as the primary outcome [29]. Notably, however, higher TISS 28 scores did correlate with the statistically significant use of critical care services. A description of the outcomes for the included studies is given in Table 2.

### 3.3. Quality Assessment

Because all studies were observational, the NOS scale was the only quality scale utilized for this analysis. The quality assessment analysis is shown in Table 3. All included studies had a score of ≥6, indicating moderate to high quality.

### 3.4. Results of Individual Studies

Albright 2014 et al. looked at the SOS at emergency department assessment as a potential model for the prediction of critical care admission up to 48 h after hospitalization, which was the primary outcome in their retrospective cohort study [28]. The cohorts were divided into those with SOS ≥ 6 or <6. Of 850 total patients, only nine patients had critical care admission, which included 16.7% from the SOS ≥ 6 group and 0.1% from the SOS < 6 group. Because the cohorts were based on the SOS score and not the ICU status, demographic and other clinical data could not be inferred regarding the critical care stay. There were no maternal deaths in their population.

Albright’s 2014 study set up the SOS prediction model for ED patients, and, in 2017, Albright et al. sought to internally validate their initial findings [31]. As such, they performed a prospective validation study of the SOS score to predict ICU admission within 48 h. The included population was 425 patients who met the sepsis criteria. Their study confirmed that those with SOS ≥ 6 had an ICU admission rate of 15%, compared to those with SOS < 6 with an ICU admission rate of 1.4%, *p* < 0.01. They calculated the association with the need for critical care admission, with sensitivity of 64%, specificity of 88%, positive predictive value of 15% and negative predictive value of 98.6%.

Agarwal et al. performed a prospective observational study to describe the SOFA score and SOS in pregnancy-associated sepsis and correlated these scores with ICU admission [32]. The mortality rate in their study was 31.7%, which was higher than in other studies. Like Albright’s studies, this study looked categorically rather than discretely at the SOFA scores. Agarwal et al. described SOFA ≥ 6 as having high significance (*p* < 0.001) regarding its correlation with both critical care admission and mortality. An SOS cutoff of ≥ 6 did not have high sensitivity (64%) or specificity (40%) for ICU admission or for maternal mortality (65% and 63%, respectively).

Minville et al. looked at hospitalized maternal populations based on specific diagnoses—pre-eclampsia, eclampsia, HELLP syndrome, acute fatty liver of pregnancy and hemolytic uremic syndrome—and compared the APACHE II, SAPS II and SOFA at admission with these diagnoses [29]. Rather than looking at critical care admission specifically, however, they correlated these findings with a measure of the nursing workload known as the TISS 28. The TISS 28 ≥ 20 group seemed to correlate highly with critical care interventions; therefore, this study was included in the analysis for the purpose of examining our primary outcomes—clinical scores and their associations with critical care needs. Patients who had TISS 28 ≥ 20 were associated with significantly higher rates of critical care procedures, *p* = 0.001. Patients whose TISS 28 ≥ 20 were also associated with longer ICU and hospital LOS values and higher rates of neonatal death.

Naz et al. looked at the SOFA score and SOS at admission to predict ICU admission in septic peripartum patients [33]. This study, like that of Agarwal, had a high mortality rate of 31.7%. The study compared SOFA versus SOS subgroups rather than discrete scores. They showed that SOFA < 6 had sensitivity and specificity for ICU admission of 84.4% and 61.3%, respectively. There was no significant difference in critical care admission for SOS < 6 or SOS ≥ 6.

Yousuf et al. performed a prospective evaluation of septic peripartum patients admitted to the ED to evaluate whether SOS predicted critical care admission [34]. In their study of 130 patients, 19 (14.6%) required ICU admission. Like most of the studies evaluating the SOS, they used a categorical cutoff of ≥6 or <6, rather than continuous numerical values. A total of three patients died in their population. Maternal death did not occur in patients with SOS < 6.

Walker et al. evaluated patients who did or did not require critical care upgrades during admission [30]. In total, 1855 peripartum patients were screened, and a total of 37 control and 34 upgrade patients were evaluated after propensity score matching. The median SOFA score was higher in ICU compared to non-upgrade patients (2 versus 0, respectively). The APACHE II score was also associated with ICU upgrades within 12 h of hospital admission.

### 3.5. Results of Syntheses

A total of 1813 peripartum patients were included. The mean age was 27.2 (±2.36). Comorbid conditions, demographic variables and vital signs were not consistently recorded or documented; therefore, they were not synthesized in this meta-analysis.

#### 3.5.1. Primary Outcome—SOFA Score

**Figure 2:**
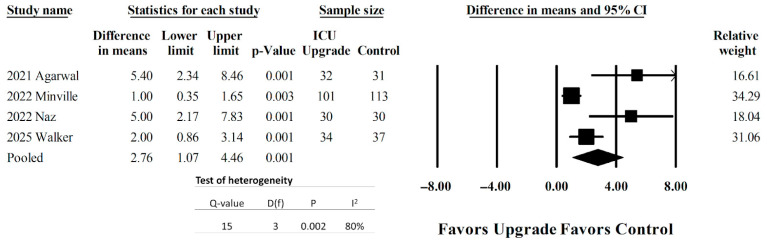
Forest plot to compare SOFA scores between patients requiring ICU upgrades and those who did not need ICU upgrades [29,30,32,33].

Four studies [29,30,32,33] reported discrete SOFA scores between the ICU upgrade and the control patients. Pooled results can be seen in Figure 2. Patients with ICU upgrades were associated with larger differences in the mean SOFA scores. The pooled difference in means was 2.76 (95% CI 1.07–4.46, *p* < 0.001). There was significant heterogeneity measured, with an I^2^ of 80%.

#### 3.5.2. Secondary Outcomes—SOS

**Figure 3:**
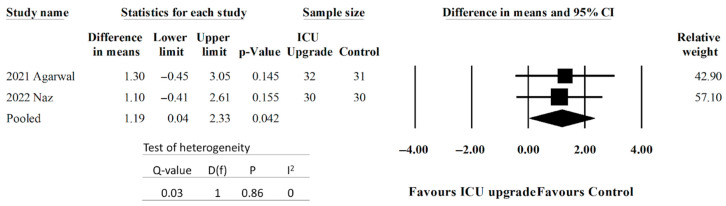
Forest plot comparing SOS scores between ICU upgrade and control populations [32,33].

Two studies reported discrete SOS scores [32,33]. Other studies did not report discrete SOS scores—for example, Albright et al. described only SOS < 6 and SOS ≥ 6 [28,31]. Pooled data can be seen in Figure 3. Patients with ICU upgrades had statistically larger differences in the mean SOS outcomes. The pooled difference in means was 1.19 (95% CI 0.04–2.33, *p* < 0.042). There was low heterogeneity.

#### 3.5.3. Secondary Outcomes—APACHE II

**Figure 4:**
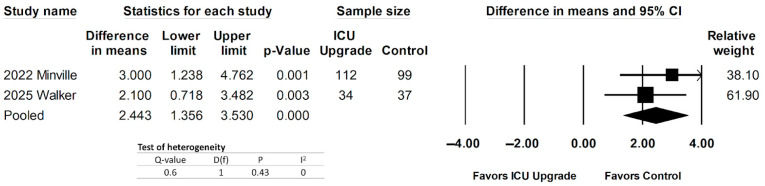
Forest plot comparing APACHE II scores between ICU upgrade and control populations [29,30].

Two studies reported APACHE II outcomes [29,30]. Pooled data can be seen in Figure 4. Patients with ICU upgrades had statistically larger differences in the mean APACHE II outcomes. The pooled difference in means was 2.43 (95% CI 1.356–3.53, *p* = 0.00). There was low heterogeneity.

#### 3.5.4. Secondary Outcomes—Shock Index

**Figure 5:**
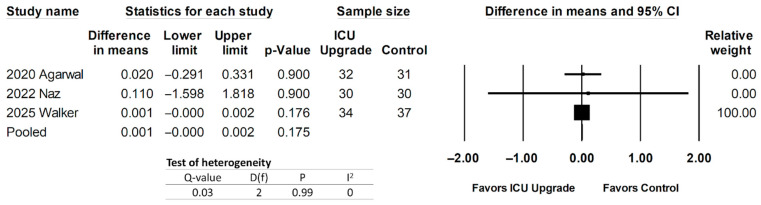
Forest plot comparing shock index between ICU upgrade and control populations [30,32,33].

The shock index was reported in one study [30], but this meta-analysis included data from two other studies [32,33] to calculate the shock index for a total of three studies analyzed regarding the outcome of the shock index. Pooled data are shown in Figure 5 and demonstrate non-significance between ICU upgrade patients and controls, with a pooled mean difference of 0.001 (95% CI −0.000–0.002, *p* = 0.175). Heterogeneity was low.

#### 3.5.5. Secondary Outcomes—Length of Stay

**Figure 6:**
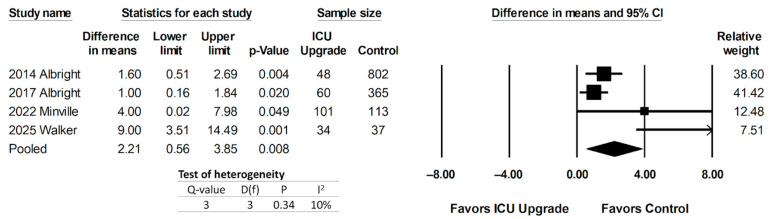
Forest plot comparing hospital length of stay (HLOS) in ICU upgrade and control populations [28,29,30,31].

Two studies evaluated the hospital length of stay (HLOS), comparing ICU upgrade to control patients [29,30]. On the other hand, both studies by Albright [28,31] compared the hospital length of stay in patients with SOS < 6 to that in patients with ≥6, which were used as a surrogate for an ICU level of care. Pooled data can be found in Figure 6. There was a significant increase in the HLOS in ICU patients or patients with SOS ≥ 6, with a pooled mean difference of 2.21 days (95% CI 0.56–3.85, *p* = 0.0.008). Heterogeneity was low, with I^2^ = 10%.

#### 3.5.6. Secondary Outcomes—Mortality

**Figure 7:**
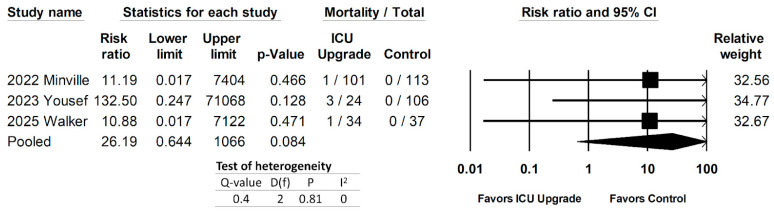
Forest plot comparing mortality rates between ICU upgrade and control patients [29,30,34].

Three studies evaluated mortality in ICU upgrade versus control patients [29,30,34]. The pooled data can be seen in Figure 7. Yousef did not qualify whether mortalities were in ICU or non-ICU patients; however, given the association of the SOS with higher organ failure and other indicators of mortality, we assumed that the patients who died were in the ICU group. There was a non-statistically significant increase in mortality in ICU patients, with a pooled risk ratio of 26.19 (95% CI 0.644–1066, *p* = 0.084). ICU-upgraded patients were associated with an overwhelmingly large odds ratio for mortality; therefore, the result was expressed as the log odds ratio.

### 3.6. Reporting Biases

Due to the small number of included studies, we did not perform moderator analyses or create a funnel plot for publication bias. However, the Egger’s and Begg’s tests for the SOFA, hospital length of stay and mortality indicated the presence of publication bias for these outcomes (Appendix C). In contrast, the analysis to compare the shock index between the two patient populations suggested that there was no publication bias for this particular outcome (Appendix C).

### 3.7. Certainty of Evidence

Given the significant heterogeneity of the studies included, our certainty of evidence is low.

## 4. Discussion

### 4.1. General Interpretation of Results in the Context of Other Evidence

To the best of our knowledge, there are no other systematic reviews looking specifically at clinical scores that are present in the ED that can help guide admission decisions in peripartum patients. Most available studies evaluate Modified EWS that occur after admission [7,8,9,10,11,12]. Comorbid conditions and diagnoses that are associated with admission to the ICU have also frequently been studied [18,19,20,21,22,23].

The majority of studies that do evaluate clinical scores at admission investigate the diagnosis of sepsis [28,31,32,33,34]. Only one study evaluated scores in the general population of peripartum patients admitted from the ED who later required critical care [30].

This study synthesized the available evidence to determine whether there is an appropriate clinical score to determine high-risk peripartum patients who may require critical care after admission, and, based on this analysis, the SOFA score could potentially be used for this purpose. The SOFA score was significantly higher in patients requiring critical care. Moreover, significantly higher SOS and APACHE II scores were present in ICU patients compared to controls. The Newcastle–Ottawa Scale evaluation of the included studies indicated that moderate- to high-quality studies were included.

### 4.2. Implications of the Results for Practice, Policy or Further Research

Higher mortality for unexpected transfers of patients in the general medical population to the ICU has previously been described [13,14,15]. It is expected that the same would hold true for peripartum patients unexpectedly requiring transfer to the ICU. If peripartum patients are identified at admission as having a higher risk for clinical decline, then admitting them to the ICU preemptively may improve their outcomes. Our study found a very large but non-statistically significant increase in mortality, and, while the confidence interval was high, it does seem plausible and consistent with other studies. In surgical patient populations, for example, the early triage of patients to critical care has demonstrated improved survival outcomes compared to patients with delayed admission to the ICU [35].

However, overtriage to the ICU also increases risky, invasive procedures, as well as costs, without necessarily improving outcomes. One interesting study evaluated four specific disease states across multiple hospitals and compared the outcomes of patients treated in the ICU and non-ICU settings [17]. The authors found that, for each condition, at a hospital level, there was no association between the ICU utilization rate and improved mortality rate; however, invasive procedures and the costs of hospitalization were increased in hospitals with increased ICU utilization.

Improvements in the outcomes of peripartum patients are essential. About 260,000 women died during and following pregnancy and childbirth in 2023 [36]. Maternal morbidity and mortality are affected by social disparities, as well as irregularities in healthcare systems. The WHO has laid out strategies to improve maternal morbidity and mortality rates, including recommendations to improve healthcare disparities [37]. The utilization of clinical scores such as the SOFA, SOS or APACHE II scores could allow for the non-discriminant determination of healthcare triage needs, rather than solely relying on sociodemographic factors or diagnoses alone.

Vital signs, symptoms and laboratory values are affected in the normal physiology of pregnancy [10,38]. The increase in metabolic demand and the need for the support of the fetus through the placenta cause a multitude of cardiovascular changes, including variations in the systemic vasculature, increases in cardiac output, decreases in mean arterial pressure and increases in heart rate [38]. The usual EWS, which rely on basic vital signs such as heart and respiratory rates, may not be as effective in identifying obstetric patients with potential for clinical decline [10]. Identifying clinical scores where true pathology and risks exist are important for patient outcomes, staff morale and financial outcomes.

## 5. Limitation

### 5.1. Limitations of the Evidence Included in the Review

Our study looked solely at observational studies; therefore, the heterogeneity was high. Due to the heterogeneity found in these observational studies, the included studies did not report information uniformly or consistently. Assumptions were made in some of our statistical analyses due to these inconsistent data. For example, Yousef did not report specific ICU or non-ICU mortality [34], but, given the representation of their population, we assumed that their mortalities occurred solely in the ICU population.

Moreover, while we utilized the Newcastle–Ottawa Scale to grade quality, the included observational studies, despite having high quality, were still at risk for biases such as patient selection. There are no methods to truly assess the bias among observational studies.

Our study also had several protocol deviations because of the significant variability in the inclusion and exclusion criteria, as well as the different timeframes within which outcomes were measured. Furthermore, we had initially intended for our population to include only patients admitted to the floor who were later upgraded to the ICU; however, the specificity of this question was too high, despite searching through thousands of articles. We therefore chose to include studies looking at clinical scores between patients, if the clinical score was obtained at admission and the ICU upgrade was within 48 h.

We included several studies that did not exactly fulfil our study protocol due to minor variations. Our protocol, for example, included patients with a gestational age greater than 20 weeks; however, the majority of our studies included any gestational age due to the lack of literature specifically looking at gestational age. Moreover, authors such as Albright and Minville had populations divided by clinical score rather than by ICU admission [28,29,31]. Because the scores correlated closely with ICU admission, however, we opted to include these studies in our review.

Meanwhile, the number of studies that included our primary outcome of the SOFA score was limited. This appears to be related to the fact that most of the included studies evaluated sepsis and the SOS score. Because most studies looked at peripartum patients with sepsis, our results may not be generalizable to all peripartum patients who might require critical care. Only one study included all types of critically ill obstetric patients [30], and one study included hypertensive disorders in pregnancy [29]. Pregnancy complications such as hemorrhage were not largely represented, and, because of this limitation in population types, our conclusions may suggest that scores are affected only in sepsis. Furthermore, we only included studies that involved patients with no previously known pregnancy complications, limiting the conclusions of this study.

### 5.2. Limitations of the Review Process Used

Due to the large number of titles and abstracts screened, we utilized several reviewers in this process. To mitigate this limitation, however, one senior investigator arbitrated any disputes over study inclusion or exclusion. We also did not include abstracts or grey literature; therefore, some smaller studies or data might have been excluded.

## 6. Conclusions

Clinical scores may be used to better triage obstetric patients to the appropriate clinical settings at admission. This systematic review and meta-analysis demonstrated higher SOFA scores in ICU upgrade patients compared to those who remained on the wards. ICU upgrade patients were also associated with a longer HLOS and higher mortality compared with control patients.

## Figures and Tables

**Table 1:** Summary of studies and patient characteristics comparing clinical scores at admission in intensive care and non-intensive care unit admissions.

Author (Year)	Country of Study	Study Design	Size (N)	Patient Subtype (Sepsis, HTN, General)	Mean Age (Years)	Included Participants	Clinical Scores Evaluated	Other Outcomes	Number of ICU Patients
Agarwal (2020) [32]	India	Prospective Cohort	63	Sepsis	26.1 ± 5.0	Inclusion Criteria: Pregnant, post-abortal (up to 2 weeks) and post-partum (≤6 weeks) obstetric patients in the age group 20–35 years with clinical sepsis fulfilling qSOFA criteria were included Exclusion Criteria: Subjects with previously known history or diagnosed pathology of pulmonary, cardiac, renal, hepato-biliary and nervous systems were excluded	SOFA, SOS, Shock Index *		32
Albright (2014) [28]	USA	Retrospective Cohort	850	Sepsis	26.17 ± 6.14 *	Inclusion Criteria: Pregnant or post-partum (≤2 weeks) patients who had blood cultures or influenza swabs sent and who presented to the ED were included Exclusion Criteria: Subjects with known or suspected ectopic pregnancy, multiple gestation, transfer from outside hospital or subsequent delivery at outside hospital were excluded	SOS	Hospital LOS	48
Albright (2017) [31]	USA	Prospective Cohort	425	Sepsis	26.59 ± 5.66 *	Inclusion Criteria: Subjects with a confirmed intrauterine pregnancy to 2 weeks post-partum who presented to the ED of the Women and Infants Hospital, a tertiary-care women’s hospital, and met modified criterion (2) with a suspected source of infection were included Exclusion Criteria: Subjects who were transferred from an outside hospital or had no suspected source of infection were excluded	SOS	Hospital LOS	60
Minville (2022) [29]	France	Retrospective Cohort	214	Hypertensive Disorders	30 ± 6 *	Inclusion Criteria: Subjects with one or more of the following diagnoses on admission in the ICU or during hospitalization were included: pre-eclampsia, eclampsia, HELLP syndrome, AFLP, HUS Exclusion Criteria: Subjects with isolated diagnoses of post-partum hemorrhage were excluded	SOFA, APACHE II, SAPS	Hospital LOS, Maternal Mortality	119
Naz (2022) [33]	Pakistan	Prospective Cohort	60	Sepsis	27 ± 2	Inclusion Criteria: Subjects 21 years or older with qSOFA criteria and clinical sepsis who were pregnant, post-abortive (up to two weeks following the operation) or post-partum (less than 6 weeks) were included Exclusion Criteria: Subjects with known history of or who had been diagnosed with pathology of the pulmonary, cardiac, renal, hepatobiliary or neurologic systems were excluded	SOFA, SOS, Shock Index *		30
Walker (2025) [30]	USA	Retrospective Case–Control	71	All Peripartum	29.5 ± 5.8	Inclusion Criteria: Subjects with greater than 20 weeks gestation or less than four weeks post-partum were included Exclusion Criteria: Subjects who were transferred from outside the ICU or admitted directly to the ICU, with a gestational age less than 20 weeks or a diagnosis of abnormal pregnancy, were excluded	SOFA, APACHE II, Shock Index	Hospital LOS, Maternal Mortality	34
Yousuf (2023) [34]	India	Prospective Cohort	130	Sepsis	30.1 ± 3.14	Inclusion Criteria: Subjects who were pregnant, post-abortal up to 2 weeks or post-partum up to 6 weeks were included Exclusion Criteria: Subjects who had non-obstetric cases of suspected sepsis or ectopic pregnancies were excluded	SOS	Maternal Mortality	19
Total *			1813		27.2 ± 2.36				342

ICU = intensive care unit; ED = emergency department; HTN = hypertension; qSOFA = Quick Sequential Organ Failure Assessment; SOFA = Sequential Organ Failure Assessment; SOS = Sepsis in Obstetrics; APACHE II = Acute Physiology and Chronic Health Evaluation II; SIRS = systemic inflammatory response syndrome; SAPS = Simplified Acute Physiology Score; LOS = length of stay; AFLP = acute fatty liver of pregnancy; HELLP = hemolysis, elevated liver enzymes and low platelet count; HUS = hemolytic uremic syndrome. * calculated values.

**Table 2:** Summary of clinical scores and outcomes in included studies.

Author (Year)	SOFA ICU	SOFA Control	SOS ICU	SOS Control	APACHE II ICU	APACHE II Control	Shock Index ICU	Shock Index Control	LOS ICU	LOS Control	Mortality ICU	Mortality Control
Agarwal (2020) [32]	10.7	5.3	8.3	7.0			1.29	1.26				
Albright (2014) [28]			SOS ≥ 6	SOS < 6								
Albright (2017) [31]			SOS ≥ 6	SOS < 6								
Minville (2022) [29]	2	1.0			7.0	4.0			15.0	11.0		
Naz (2022) [33]	10.4	5.4	8.2	7.1			1.26	1.16				
Walker (2025) [30]	1.8	0.25			5.2	3.1	0.80	0.80	12.7	3.7	1	0
Yousuf (2023) [34]			SOS ≥ 6	SOS < 6							3	0

SOFA = Sequential Organ Failure Assessment; SOS = Sepsis in Obstetrics Score; ICU = intensive care unit; APACHE II = Acute Physiology and Chronic Health Evaluation II; LOS = length of stay.

**Table 3:** Quality assessment utilizing the Newcastle–Ottawa Scale.

Author (Year)	Study Design	Selection—1	Selection—2	Selection—3	Selection—4	Comparability—1	Outcome—1	Outcome—2	Outcome—3	Total—Selection	Total—Comparability	Total—Outcome	Total Score
Agarwal (2020) [32]	Cohort Study	0	1	1	1	0	1	1	1	3	0	3	6
Albright (2014) [28]	Cohort Study	0	1	1	1	2	1	1	1	3	2	3	8
Albright (2017) [31]	Cohort Study	0	1	1	1	2	1	1	1	3	2	3	8
Minville (2022) [29]	Cohort Study	0	1	1	1	2	1	1	1	3	2	3	8
Naz (2022) [33]	Cohort Study	0	1	1	1	0	1	1	1	3	0	3	6
Walker (2025) [30]	Case– Control Study	1	0	0	1	2	1	1	1	2	2	3	7
Yousef (2023) [34]	Cohort Study	0	1	1	1	1	1	1	1	3	1	3	7

## Data Availability

No new data were created or analyzed in this study. All data in this study were from publicly available sources.

## Appendix A

**Table A1:** Documentation of search terms and strategies performed.

#1	Critical care [Mesh] OR Intensive care units [Mesh] OR critical-care [tiab] OR intensive-care [tiab] OR ICU [tiab] OR “ICU upgrade” [tiab:~4] OR “ICU transfer” [tiab:~4] OR critical-illness [tiab] OR icu-admission [tiab] OR obstetric-icu [tiab] OR obstetric-intensive [tiab]
#2	Pregnancy [Mesh] OR pregnancy complications [Mesh] OR peripartum period [Mesh] OR Gestational Age [Mesh] OR pregnan * [tiab] OR peripartum [tiab] OR obstetric [tiab] OR pregnancy-related [tiab] OR perinatal [tiab] OR pregnancy-complication * [tiab] OR maternal [tiab] OR gestation * [tiab]
#3	(“Hypertension, Pregnancy-Induced” [Mesh] OR preeclampsi * [tiab] OR eclampsia * [tiab] OR “pregnancy induced hypertension” [tiab] OR “pregnancy induced hypertensions” [tiab] OR toxemi * [tiab] OR “gestational hypertension” [tiab] OR pih [tiab] OR eph [tiab] OR (gestosis [tiab] AND hypertensi * [tiab]) OR (transient [tiab] AND hypertensi * [tiab] AND pregnan * [tiab]))
#4	Risk factors [Mesh] OR risk-factor * [tiab] OR characteristic * [tiab] OR indication * [tiab] OR profile * [tiab] OR baseline [tiab] OR indicator * [tiab] OR predict * [tiab] OR epidemiology [tiab] OR pregnancy complications [Mesh] OR pregnancy-complication * [tiab] OR maternal-complication * [tiab]
#5	#1 AND (#2 OR #3) AND #4
	Ovid Medline
1	(exp critical care/or exp intensive care units/or (ICU or ((critical or intensive or ICU) adj3 (care or unit or hospital) adj3 (admission * or transfer * or upgrade))).ti,ab.)
2	(pregnancy/or exp pregnancy complications/or peripartum period/or gestational age/or (pregnan * or gestational or perinatal or peripartum or maternal or ((peripartum or pregnancy or maternal or peri-partum or obstetric) adj3 (complication * or emergenc * or admission))).ti,ab.)
3	((before or during or “immediately after” or “week after”) adj3 (gestation or child-birth or childbirth or labor or delivery)).ti,ab.
4	(complication * or emergenc* or admission or hospital * or transfer * or upgrade))).ti,ab.)
5	(exp risk factors/or (risk-factor * or characteristic * or indication * or profile * or indicator * or predict * or epidemiology or clinical-factor* or risk-factor*).ti,ab.)
6	2 or 3
7	4 or 5
8	1 and 6 and 7

* = Truncation used for variant endings for a term.

## Appendix B

**Table A2:** Summary of study protocol adherence and deviation categories.

Author (Year)	Included Participants	Time to ICU Admission from ED	Protocol Deviation (Admission Location)	Protocol Deviation (Patient Age)	Protocol Deviation (Gestational Age)	Protocol Deviation (Abnormal Pregnancy)
Albright et al. (2014) [28]	Peripartum patients with sepsis	At admission up to 48 h	ICU admission/transfer within 48 h to ICU	Included age < 18 years old	Any gestational age but ≤2 weeks post-partum	None
Albright et al. (2017) [31]	Peripartum patients with sepsis	At admission up to 48 h	Direct ICU admissions	None	Any gestational age up to 2 weeks post-partum	Not specified
Agarwal et al. (2021) [32]	Peripartum patients with sepsis	At admission	Direct ICU admissions	None	Any gestational age up to ≤6 weeks	Not specified
Naz et al. (2022) [33]	Peripartum patients with sepsis	At admission	Direct ICU admissions	None	Any gestational age, post-abortive or ≤6 weeks	Not specified
Minville et al. (2020) [29]	Peripartum patients with pre-eclampsia, eclampsia, HELLP syndrome, AFLP and/or HUS	At admission	Compared TISS 28 ≥ 20 and TISS 28 < 20 as a marker of nursing workload	None	None	Not specified
Yousuf et al. (2023) [34]	Peripartum patients with sepsis	At admission	Direct ICU admissions	None	Any gestational age, post-abortal or ≤6 weeks	Not specified
Walker et al. (2025) [30]	All peripartum patients	At admission	ICU upgrade versus no upgrade	None	None	None

Abbreviations: ICU = intensive care unit, ED = emergency department, HELLP = hemolysis, elevated liver enzymes and low platelets, AFLP = acute fatty liver of pregnancy, HUS = hemolytic uremic syndrome, TISS = Therapeutic Intervention Scoring System.

## Appendix C

**Table A3:** Results from Egger’s and Begg’s tests for publication biases among the outcomes. Both tests were only performed for outcomes that included three or more studies. Any *p*-value < 0.05 indicates the presence of publication bias.

Outcome	Number of Included Studies	*p*-Value		Presence of Publication Bias
		Egger’s Test	Begg’s Test	
SOFA	4	0.089	0.008	Yes
SOS	2	NA	NA	NA
APACHE II	2	NA	NA	NA
Shock index	3	0.601	0.199	No
Hospital length of stay	4	0.041	0.046	Yes
Mortality	3	0.0006	0.089	Yes

APACHE, Acute Physiological Assessment and Chronic Health Evaluation; NA, analyses were not performed due to the small number of studies being included; SOFA, Sequential Organ Failure Assessment; SOS, Sepsis in Obstetrics Score.
